# Phytochrome phosphorylation in plant light signaling

**DOI:** 10.3389/fpls.2024.1259720

**Published:** 2024-03-12

**Authors:** Yun-Jeong Han, Seong-Hyeon Kim, Jeong-Il Kim

**Affiliations:** ^1^ Kumho Life Science Laboratory, Chonnam National University, Gwangju, Republic of Korea; ^2^ Department of Integrative Food, Bioscience and Biotechnology, Chonnam National University, Gwangju, Republic of Korea

**Keywords:** plant phytochromes, phosphorylation, protein kinase, dephosphorylation, light signaling

## Abstract

Plant phytochromes, renowned phosphoproteins, are red and far-red photoreceptors that regulate growth and development in response to light signals. Studies on phytochrome phosphorylation postulate that the N-terminal extension (NTE) and hinge region between N- and C-domains are sites of phosphorylation. Further studies have demonstrated that phosphorylation in the hinge region is important for regulating protein–protein interactions with downstream signaling partners, and phosphorylation in the NTE partakes in controlling phytochrome activity for signal attenuation and nuclear import. Moreover, phytochrome-associated protein phosphatases have been reported, indicating a role of reversible phosphorylation in phytochrome regulation. Furthermore, phytochromes exhibit serine/threonine kinase activity with autophosphorylation, and studies on phytochrome mutants with impaired or increased kinase activity corroborate that they are functional protein kinases in plants. In addition to the autophosphorylation, phytochromes negatively regulate PHYTOCHROME-INTERACTING FACTORs (PIFs) in a light-dependent manner by phosphorylating them as kinase substrates. Very recently, a few protein kinases have also been reported to phosphorylate phytochromes, suggesting new views on the regulation of phytochrome via phosphorylation. Using these recent advances, this review details phytochrome regulation through phosphorylation and highlights their significance as protein kinases in plant light signaling.

## Introduction

As sessile organisms, searching for light is imperative for the optimal growth and development of higher plants. Thus, they evolved multiple photoreceptors, including red (R) and far-red (FR) light-sensing phytochromes encoded by small gene families (Inoue et al., 2017; Rockwell and Lagarias, 2020). For example, among the five family members in *Arabidopsis thaliana* (phyA to phyE), phyA and phyB are integral for FR and R light signaling (Mathews, 2010; Sheerin and Hiltbrunner, 2017; Kim et al., 2021). It is also notable that phyA is light-labile, whereas phyB–phyE are relatively light-stable. These phytochromes serve as molecular switches to translate light signals into physiological responses of plants by photocycling between two photoisomers: R light-absorbing Pr (switched off or inactive form) and FR light-absorbing Pfr (switched on or active form). Phytochromes in higher plants are biosynthesized as Pr, and transformed into Pfr upon light exposure. This photoactivation regulates plant photomorphogenic development via highly regulated signaling networks (Cheng et al., 2021).

**Figure 1:**
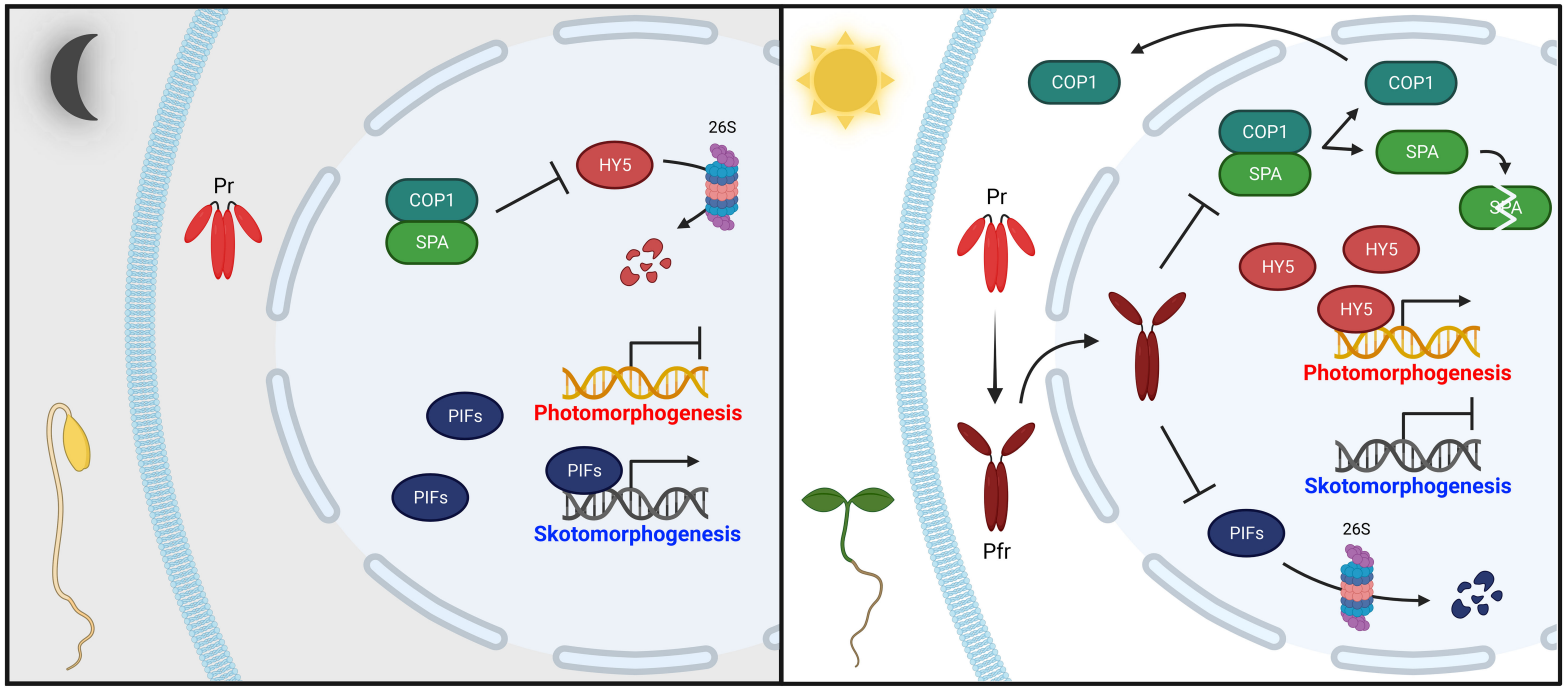
Simplified phytochrome-mediated regulatory mechanisms for photomorphogenesis in seedlings. In the dark (left panel), phytochromes are biosynthesized as inactive Pr in the cytoplasm. Concurrently, positive photomorphogenesis regulators, such as HY5, are degraded by the COP1/SPA complex via the 26S proteasome pathway, whereas negative regulators, such as PIFs, sustain skotomorphogenic development by the repression of photomorphogenesis (i.e., etiolated seedlings). In the light (right panel), photoactivated phytochromes (Pfr) move from the cytoplasm into the nucleus, where they negatively regulate PIFs and the COP1/SPA complex. PIFs are mostly degraded through the 26S proteasome pathway. The COP1/SPA complex is dissociated, prompting COP1 nuclear exclusion and SPA protein degradation. As a result, accumulated HY5 promotes photomorphogenic development (i.e., de-etiolated seedings). Abbreviations are listed in **Supplementary Table S1**.

Since the discovery of phytochromes, extensive efforts have been made to elucidate how they mediate plant light signaling (Legris et al., 2019). As a result, various phytochrome-interacting proteins have been identified and their functions have been studied (Bae and Choi, 2008; Pham et al., 2018). Essentially, a fundamental regulatory mechanism for phytochrome signaling is the transcriptional regulation of photoresponsive genes by promoting the degradation or inactivation of negative regulators, such as PHYTOCHROME-INTERACTING FACTORs (PIFs), and the resulting accumulation of positive regulators, such as ELONGATED HYPOCOTYL 5 (HY5) (**Figure 1**). In the dark, PIFs are accumulated, and HY5 is degraded by an E3 ubiquitin ligase complex comprising CONSTITUTIVE PHOTOMORPHOGENIC 1 (COP1) and SUPPRESSORs OF *phyA-105* (SPAs) (Hoecker, 2017; Xiao et al., 2021; Wang et al., 2022). Thus, PIFs promote skotomorphogenesis by repressing photomorphogenesis. This skotomorphogenic development prompts etiolated seedlings showing long hypocotyls and closed cotyledons with apical hooks (**Figure 1**, left). In the light, photoactivated phytochromes move from the cytoplasm into the nucleus, where they interact with downstream signaling partners to promote photomorphogenesis (Fankhauser and Chen, 2008; Klose et al., 2015; Helizon et al., 2018). Notably, phytochrome interactions with PIFs are pivotal to catalyzing their degradation via the ubiquitin/26S proteasome pathway (Favero, 2020). Concurrently, phytochromes also induce dissociation of the COP1/SPA complex, effectuating COP1 nuclear exclusion and SPA protein degradation (Subramanian et al., 2004; Chen et al., 2015; Lu et al., 2015; Sheerin et al., 2015; Podolec and Ulm, 2018). When the COP1/SPA complex is inactivated, HY5 is accumulated and promotes photomorphogenic development by controlling transcription of one-third of all genes in plants (Jing and Lin, 2020; Xiao et al., 2021). This regulation generates de-etiolated seedlings through inhibition of hypocotyl elongation, chloroplast differentiation with chlorophyll accumulation, and cotyledon expansion (**Figure 1**, right). Phytochromes also participate in many other stages of plant growth and development (Song et al., 2020; Choi et al., 2023).

Although the physiological functions of phytochromes and their signaling partners in plants are relatively well-studied, the molecular and regulatory mechanisms for phytochrome signaling have yet to be fully elucidated. Historically, phytochromes have been identified as phosphoproteins (Hunt and Pratt, 1980); therefore, protein kinases (PKs) and protein phosphatases (PPases) may be involved in phytochrome signaling. Moreover, purified phytochromes displayed serine/threonine PK activity, suggesting them as autophosphorylating PKs (Yeh and Lagarias, 1998). Furthermore, a few PPases have been discovered to be phytochrome-associated (Kim et al., 2002; Ryu et al., 2005; Phee et al., 2008), and further studies report that phytochromes regulate plant light signaling by acting as functional PKs (Shin et al., 2016; Hoang et al., 2021). Thus, it is apparent that phosphorylation and dephosphorylation are involved in phytochrome signaling. Although a recent review describes the regulation of plant photomorphogenesis by the PK activity of phyA (Choi et al., 2023), it is worthwhile to review phytochrome phosphorylation in plant light signaling by focusing not only on phyA but also on other phytochromes including phyB, with recent studies on PKs that can phosphorylate phyB (Liu et al., 2023; Zhao et al., 2023). Therefore, this review highlights regulation of phytochromes through phosphorylation and dephosphorylation, and their roles as PKs in plant light signaling.

## Phytochrome regulation through phosphorylation and dephosphorylation

**Figure 2:**
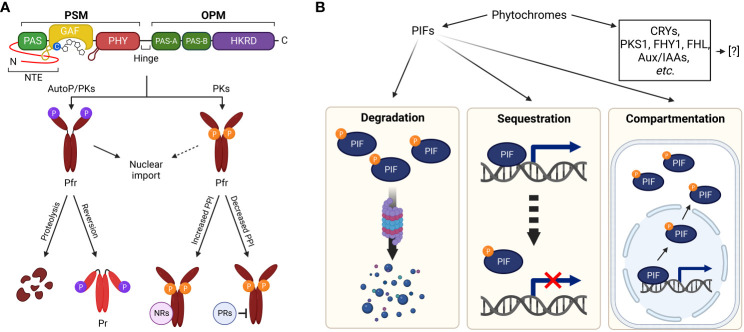
Plant light signaling regulated by phytochromes via phosphorylation. **(A)** Phytochrome regulation via phosphorylation. The NTE and hinge region between PSM and OPM are phosphorylated by phytochromes (AutoP) and/or PKs. The PSM contains a photosensory core (PAS-GAF-PHY) with a tetrapyrrole chromophore bound to a conserved cysteine residue. A well-ordered hairpin in the PHY and light-sensing knot lasso motif at the PAS-GAF interface are also shown. The OPM includes two PAS domains (PAS-A and PAS-B) and an HKRD. The NTE phosphorylation in Pfr forms of phyA and phyB induces rapid degradation and accelerated reversion to Pr, respectively. Hinge region phosphorylation regulates protein–protein interactions between phytochromes and negative regulators (NRs, for increased interactions) or positive regulators (PRs, for decreased interactions). It is also reported that phytochrome phosphorylation is involved in the regulation of nuclear import. **(B)** Regulation of downstream signaling partners via phytochrome kinase activity. As PKs, phytochromes phosphorylate substrate proteins, such as CRYs, PKS1, Aux/IAAs, FHY1/FHL, and PIFs. Besides PIFs, the regulatory mechanisms of phytochromes for the substrate proteins have yet to be fully elucidated. Concerning the phosphorylation of PIFs by phytochromes, 26S proteasome-mediated degradation (left), sequestration from target promoters (middle), and compartmentalization to the cytoplasm (right) have been demonstrated as the regulatory mechanisms. Abbreviations are listed in **Supplementary Table S1**.

A previous phosphate content analysis with immunoaffinity-purified *Avena sativa* phyA (AsphyA) determined approximately one phosphate per phytochrome monomer (Hunt and Pratt, 1980). In addition, oat and maize phytochrome phosphorylation was demonstrated *in vitro* with PKs (Wong et al., 1986; Biermann et al., 1994). Extensive studies have endeavored to locate phosphorylation sites, and three serine residues have been identified using AsphyA: S8, S18, and S599 (Lapko et al., 1999). Phytochromes are dimers, and each monomer comprises an N-terminal photosensory module (PSM) and a C-terminal output module (OPM) connected by a hinge region (**Figure 2A**). In the PSM, a PAS-GAF-PHY tri-domain, also known as the photosensory core for absorbing light, is responsible for the bilin lyase activity of phytochromes to bind a tetrapyrrole chromophore to a conserved cysteine residue (Wahlgren et al., 2021; Li et al., 2022). In addition, an N-terminal extension (NTE; 1–65 aa region of AsphyA) has been reported to be essential for biological activity (Cherry et al., 1992). Recently, NTE has also been suggested as an intrinsically disordered region that modulates liquid–liquid phase separation of phytochromes (Chen et al., 2022). In the OPM, there are two PAS domains (PAS-A and PAS-B) and a histidine kinase-related domain (HKRD), which influences dimerization, nuclear localization, and protein–protein interactions with signaling partners (Qiu et al., 2017; Cheng et al., 2021). Thus, the phosphorylation site analysis of AsphyA proposes that phytochromes could be phosphorylated at the NTE and the hinge region (**Figure 2A**).

Among the phosphorylation sites of AsphyA, the S599 in the hinge region is phosphorylated in a Pfr-specific manner (Lapko et al., 1999). A previous study concluded that transgenic lines with S599A mutation exhibited hypersensitive responses to FR light, proposing an inhibitory role of the hinge region phosphorylation (Kim et al., 2004). In addition, the S599A protein interacted more potently with NUCLEOSIDE DIPHOSPHATE KINASE 2 (NDPK2), a positive regulator in the phytochrome signaling (Choi et al., 1999), than wild-type AsphyA. Furthermore, PHYTOCHROME-ASSOCIATED PROTEIN PHOSPHATASE 2C (PAPP2C) affected interactions between phytochromes and PIF3, a negative regulator in the phytochrome signaling (Phee et al., 2008). It is notable that there is no homologous site to S599 of AsphyA in *A. thaliana* phyA (AtphyA). However, three serine/threonine phosphorylation sites have been identified in the hinge region of AtphyA: S590, T593, and S602 (Zhou et al., 2018). Mutating these sites impaired AtphyA function by affecting interactions with FAR-RED ELONGATED HYPOCOTYL 1 (FHY1) and FHY1-LIKE (FHL) shuttle proteins for the nuclear import of AtphyA. Therefore, hinge region phosphorylation likely regulates protein–protein interactions between phytochromes and their signaling partners (**Figure 2A**).

Both S8 and S18 in the NTE of AsphyA were confirmed as autophosphorylation sites and transgenic plants with Ser-to-Ala mutations were hypersensitive to FR light due to significantly slower light-induced degradation rates than wild-type AsphyA (Han et al., 2010a). These results suggest that NTE phosphorylation may desensitize or attenuate phyA signaling after its activation upon light exposure (Han et al., 2010b). Consistently, the autophosphorylation sites of *A. thaliana* phyB (AtphyB) were proven to reside in the NTE (Phee et al., 2008). Subsequently, the phosphorylation sites of AtphyB have been identified, especially at S80, S86, Y104, and S106 (Medzihradszky et al., 2013; Nito et al., 2013; Viczian et al., 2020; Zhao et al., 2023). In particular, phosphorylation at S86 accelerated dark reversion (i.e., light-independent conversion of Pfr to Pr) that attenuates AtphyB function (Viczian et al., 2020). In the same study, phosphorylation of serine residues in the NTE of AtphyD (S79 or S82) and AtphyE (S53) was revealed by LC-MS/MS analyses, which are in close proximity to the conserved S86 of AtphyB. It is noted that S88 in AtphyD and S50 in AtphyE are homologous sites to S86 of AtphyB. Further studies showed that nonphosphorylatable phyD mutants (S82A and S88A) displayed hypersensitive responses to R light, whereas the phosphomimic phyD (S88D) and phyE (S50D) mutants exhibited reduced or almost blind responses to R light, respectively. Thus, these results indicate that phosphorylation at the NTE would be a general mechanism to attenuate light sensitivity of phytochromes. This is consistent with a recent report that AtphyB phosphorylation by FERONIA (FER) accelerated dark reversion with decreases in protein abundance, especially in the nucleus (Liu et al., 2023). As a note, dark reversion is also known as thermal reversion because raising ambient temperatures can accelerate the Pfr-to-Pr conversion (Klose et al., 2020). Thus, NTE phosphorylation likely promotes protein degradation (for phyA) or accelerates dark/thermal reversion (for phyB) for signal desensitization or attenuation. More recently, two calcium-dependent PKs, CPK6 and CPK12 (CPK6/12), were reported to phosphorylate AtphyB at S80 and S106 (Zhao et al., 2023). In the study, the R light-stimulated cytosolic calcium increases stimulated the phosphorylation of AtphyB by CPK6/12, which is required for phyB nuclear import in etiolated seedlings. Therefore, the NTE phosphorylation of phyB is necessary not only for accelerated dark/thermal reversion but also for light-dependent nuclear import (**Figure 2A**).

Since phytochromes are autophosphorylated or can be phosphorylated by PKs, PPases likely participate in phytochrome signaling. A PPase designated as FLOWER-SPECIFIC PHYTOCHROME-ASSOCIATED PROTEIN PHOSPHATASE (FyPP) encodes a catalytic protein phosphatase 6 (PP6) subunit and efficiently dephosphorylates autophosphorylated AsphyA in a Pfr-dependent manner (Kim et al., 2002). Transgenic plants with increased or decreased FyPP levels displayed delay or acceleration of flowering, respectively, suggesting the importance of phytochrome dephosphorylation by PP6 for the regulation of flowering. Further studies identified PHYTOCHROME-ASSOCIATED PROTEIN PHOSPHATASE 5 (PAPP5), another PPase that positively regulate phytochrome signaling in plants (Ryu et al., 2005). The protein stability of phyA and the protein–protein interaction with NDPK2 increased in *PAPP5*-overexpressing plants but decreased in *papp5* mutants, suggesting that dephosphorylation is crucial for regulating phytochrome stability and binding to signaling partners. In addition, PAPP2C is a PPase that dephosphorylates phyA and phyB, positively influencing phytochrome-mediated photoresponses in plants (Phee et al., 2008). The studies of these PPases suggest phytochrome regulation through reversible phosphorylation. For example, NTE phosphorylation by phytochromes themselves or PKs reduces Pfr levels (via rapid degradation of phyA and accelerated dark reversion of phyB), decreasing phytochrome activity, whereas its dephosphorylation intensifies the activity. Hinge region phosphorylation by PKs and dephosphorylation by PPases may also regulate protein–protein interactions between phytochromes and their signaling partners (**Figure 2A**).

Other studies also support phosphorylation and dephosphorylation of phytochromes, for example, by the identification of phyA′ and phyA′′ isoforms in plants (Sineshchekov and Koppel, 2022), which differ in post-translational modifications (phyA′ as the phosphorylated form and phyA′′ as the dephosphorylated form). Consistently, the treatment of PPase inhibitor diminished the levels of phyA′ while concomitantly elevating phyA′′ (Sineshchekov et al., 2013). Moreover, phosphorylated and unphosphorylated phytochromes interact differently with their signaling counterparts (Saijo et al., 2008). Phosphorylated phyA interacts better with the COP1/SPA complex for degradation, whereas the unphosphorylated form preferentially associates with FHY1 and FHY3 for nuclear import. These results demonstrate that phyA phosphorylation acts as a molecular switch to control differential protein–protein interactions for signal attenuation or amplification, corroborating hinge region phosphorylation properties in our model (**Figure 2A**).

## Plant light signaling regulated by the protein kinase activity of phytochromes

In the initial stage of phytochrome research, its enzymatic activity has been highly investigated, because phytochromes can interact with signaling partners in an enzyme–substrate relationship. Initial studies have demonstrated the bilin lyase activity of phytochromes for chromophore attachment (Lagarias and Lagarias, 1989), but this is not related to the interaction or regulation of signaling partners. Later, the autophosphorylating serine/threonine PK activity was demonstrated with purified recombinant phytochrome proteins *in vitro* (Yeh and Lagarias, 1998). Then, further experiments were performed to define phytochromes as functional PKs, such as mapping the kinase domain with ATP binding, identification of substrate proteins that can be phosphorylated by phytochromes, and acquisition of supporting data that the kinase activity of phytochromes is required for regulating plant light signaling.

As for the kinase domain in phytochromes, previous studies questioned whether the C-terminal HKRD was functional, as it is the only PK-related domain. However, the HKRD was suggested to be a non-functional kinase domain because residue mutations for ATP binding did not affect phytochrome function (Boylan and Quail, 1996). In addition, HKRD deletion did not abolish phyB activity in plants, and N-terminal PSM was enough to trigger full phyB activity when dimerized and localized in the nucleus (Krall and Reed, 2000; Matsushita et al., 2003). Thus, the kinase domain of phytochromes might reside in a region other than the HKRD. Later, kinase domain mapping experiments were conducted with truncated phytochromes, demonstrating that the photosensory core (PAS-GAF-PHY tri-domain; 66–610 aa region of AsphyA) displayed the observed kinase activity in plant phytochromes (Shin et al., 2016). ATP binding to the photosensory core was also verified, indicating that the ATP-binding region resides in the PHY domain. Therefore, the PAS-GAF-PHY photosensory core is now considered to be responsible for the phytochrome kinase activity.

After the PK activity of plant phytochromes was reported with histone H1 as a substrate (Yeh and Lagarias, 1998), several proteins were also identified as being phosphorylated by phytochromes. For example, CRYPTOCHROMEs (CRYs), PHYTOCHROME KINASE SUBSTRATE 1 (PKS1), and AUXIN/INDOLE-3-ACETIC ACID proteins (Aux/IAAs) were phosphorylated by phytochromes (Ahmad et al., 1998; Fankhauser et al., 1999; Colon-Carmona et al., 2000). Furthermore, phyA phosphorylated FHY1 and FHL in an R/FR light-reversible manner: R light-induced phosphorylation of PHY1 at position S39 and T61 inhibited phyA nuclear import in plants (Shen et al., 2009; Chen et al., 2012). However, how these substrates are regulated through phosphorylation by phytochromes has yet to be elucidated (**Figure 2B**). In contrast, photoactivated phyA and phyB rapidly phosphorylate PIF1, PIF3, PIF4, and PIF5 in plants, preceding 26S proteasome-mediated degradation (Al-Sady et al., 2006; Shen et al., 2007, 2008). It should be noted that autophosphorylation and histone H1 phosphorylation by phytochromes (phyA, phyB, and phyD) were significantly reduced in the presence of PIF3 as a substrate (Shin et al., 2016). These results suggested that PIF3 is a favorable substrate phosphorylated by phytochromes over phytochrome itself or histone H1. Therefore, PIFs are suggested as genuine substrates that can be phosphorylated by phytochromes.

When studying phytochrome signaling, a predominant question is the early signaling event or how phytochromes regulate their downstream signaling partners. Phosphorylation of substrate proteins by the kinase activity of phytochromes may represent the primary signaling after photoactivation. Accordingly, AsphyA mutants with altered kinase activities were obtained, and their functions were analyzed using transgenic plants. The transgenic plants expressing three mutants with decreased kinase activity (K411L, T418D, and D422R) were hyposensitive to FR light, whereas those expressing two mutants with increased kinase activity (K411R and T418V) were hypersensitive (Shin et al., 2016; Hoang et al., 2021). Moreover, FR-induced phosphorylation and degradation of PIF1 and PIF3 were positively corelated with the levels of phytochrome kinase activity. Therefore, phytochromes are now believed to function as PKs for inactivating PIFs via phosphorylation (**Figure 2B**).

When phosphorylated by phytochromes or other PKs, most PIFs are degraded by the 26S proteasome pathway (**Figure 2B**, left). In addition, phytochromes inhibited the binding of PIFs to target promoters (sequestration; **Figure 2B**, middle) (Park et al., 2012, 2018). Furthermore, PIF7 phosphorylation by phytochromes is necessary for controlling its subcellular localization, not degradation (Huang et al., 2018; Fiorucci et al., 2020; Burko et al., 2022). PIF7 is dephosphorylated in the dark or shade, resulting in its nuclear accumulation, whereas under light conditions, its phosphorylation results in its retaining in the cytoplasm (compartmentalization; **Figure 2B**, right). Therefore, recent studies propose the regulatory properties of phytochromes on PIFs as the phosphorylation via their intrinsic kinase activities, resulting in proteasomal degradation, sequestration from target promoters, and compartmentalization into the cytoplasm (**Figure 2B**).

## Discussion and perspectives

Reversible phosphorylation is a prominent and ubiquitous post-translational modification for nearly all cellular activities. Approximately 47% of expressed proteins are phosphorylated in *Arabidopsis*, as revealed by mass spectrometry (Mergner et al., 2020). Plant phytochromes are excellent examples of proteins regulated through reversible phosphorylation. As illustrated in **Figure 2A**, NTE phosphorylation attenuates phytochrome function via proteolysis (for phyA) and accelerated reversion to Pr (for phyB), whereas hinge region phosphorylation regulates protein–protein interactions with downstream signaling partners. Additionally, phytochrome phosphorylation plays roles in light-dependent nuclear import. In contrast, phytochrome-associated PPases positively influence plant light signaling, indicating that dephosphorylation acts antagonistically against phosphorylation. Concerning this regulation, PKs are necessary for phytochrome phosphorylation, especially in the NTE and hinge regions. While phytochromes can autophosphorylate the NTE (Phee et al., 2008; Han et al., 2010a), they do not phosphorylate the hinge region, suggesting the necessity of other PKs. Accordingly, FER and CPK6/12 were recently reported as the PKs that phosphorylated phyB (Liu et al., 2023; Viczian and Nagy, 2023; Zhao et al., 2023). However, the PK that phosphorylates phyA has yet to be identified. Therefore, the PKs that phosphorylate phytochromes are necessary to be identified further to elucidate phytochrome regulation through reversible phosphorylation.

PIFs are integral for phytochrome signaling as they repress various photomorphogenic responses through transcriptional regulation of over a thousand genes (Pham et al., 2018; Jing and Lin, 2020). Thus, plant growth and development are likely regulated by phytochrome-PIF signaling modules comprising five phytochromes and eight PIFs in *Arabidopsis* (Wang et al., 2022; Choi et al., 2023). For example, photoactivated phytochromes induce phosphorylation and degradation of PIF1 and PIF3 in plants, which positively regulates seed germination and de-etiolation responses, respectively. Therefore, positive regulator (i.e., phytochromes) and negative regulator (i.e., PIFs) pairs for photomorphogenesis could regulate various aspects of plant growth and development through phosphorylation of PIFs by phytochromes (**Figure 2B**). Besides PIFs, other proteins such as CRYs, PKS1, Aux/IAAs, and FHY1/FHL have also been phosphorylated by phytochromes, establishing them as substrate protein candidates. However, the effects of phosphorylation in their functions are not fully elucidated. Thus, the regulatory mechanisms on these substrate proteins through the kinase activity of phytochromes need to be studied further, including additional identification of substrate proteins. Coincidentally, primary and secondary phyB-interacting proteins in nuclear photobodies have been analyzed recently (Kim et al., 2023). As such, more substrate proteins that can be phosphorylated by phyB could be isolated from the primary interacting proteins in the study.

The presence of a kinase domain in the N-terminal PSM is interesting, because there is no sequence homology to a known PK. Previously, it was shown that the PSM is enough to exert phytochrome function when dimerized and localized in the nucleus (Matsushita et al., 2003; Viczian et al., 2012). Thus, it is believed that the PSM is essential for phytochrome function, whereas the C-terminal OPM plays roles in the regulation of phytochrome signaling. In this regard, it should be noted that deletion of the OPM increases the kinase activity of phytochromes (Shin et al., 2016). In addition, the OPM is necessary for PIF3 degradation and early light signaling (Park et al., 2012; Qiu et al., 2017). Thus, the functional roles of the OPM necessitates additional studies by focusing on the regulation of phytochrome kinase activity. Moreover, the regulation of phytochrome phosphorylation may have potentials for biotechnological applications, including optogenetics for manipulating biological activities with light (Konrad et al., 2023). Therefore, understanding the molecular and regulatory mechanisms of phytochromes via reversible phosphorylation and their kinase activity will bring further insights into the broader signaling networks underlying plant light perception and adaptation.

## Author contributions

Y-JH: Conceptualization, Funding acquisition, Investigation, Writing – original draft, Writing – review & editing. S-HK: Conceptualization, Investigation, Visualization, Writing – review & editing. J-IK: Conceptualization, Funding acquisition, Investigation, Supervision, Visualization, Writing – original draft, Writing – review & editing.
