# Supplementary material for: Phytochrome phosphorylation in plant light signaling
Source: Front Plant Sci. 2024 Mar 12;15:1259720. doi: 10.3389/fpls.2024.1259720 (PMC10967025; doi:10.3389/fpls.2024.1259720)
Supplement: Supplementary file 1 [file Table_1.pdf]

Supplementary Table S1. Abbreviations used in the present study.

**Abbreviations**	**Full name**
AutoP	Autophosphorylation
Aux/IAAs	AUXIN/INDOLE-3-ACETIC ACID proteins
AtphyA	*Arabidopsis thaliana* phytochrome A
AtphyB	*Arabidopsis thaliana* phytochrome B
AsphyA	*Avena sativa* phytochrome A
COP1	CONSTITUTIVE PHOTOMORPHOGENIC 1
CPK	Calcium-dependent protein kinase
CRYs	CRYPTOCHROMEs
FER	FERONIA
FHL	FHY1-LIKE
FHY1	FAR-RED ELONGATED HYPOCOTYL 1
FR	Far-red
FyPP	FLOWER-SPECIFIC PHYTOCHROME-ASSOCIATED PROTEIN PHOSPHATASE
GAF	cGMP PHOSPHODIESTERASE/ADENYLYL CYCLASE/FHLA
HKRD	Histidine kinase-related domain
HY5	ELONGATED HYPOCOTYL 5
NDPK2	NUCLEOSIDE DIPHOSPHATE KINASE 2
NRs	Negative regulators
NTE	N-terminal extension
OPM	Output module
PAPP2C	PHYTOCHROME-ASSOCIATED PROTEIN PHOSPHATASE 2C
PAPP5	PHYTOCHROME-ASSOCIATED PROTEIN PHOSPHATASE 5
PAS	PER/ARNT/SIM
Pfr	Far-red light-absorbing form of phytochromes
PHY	PHYTOCHROME-SPECIFIC domain
PIFs	PHYTOCHROME-INTERACTING FACTORs
PKs	Protein kinases
PKS1	PHYTOCHROME KINASE SUBSTRATE 1
PP6	Protein phosphatase 6
PPases	Protein phosphatases
Pr	Red light-absorbing form of phytochromes
PRs	Positive regulators
PSM	Photosensory module
R	Red
SPAs	SUPPRESSORs OF *phyA-105*

*Abbreviations are listed in an alphabetical order.
